# Survival comparison of first-line treatment regimens in patients with braf-mutated advanced colorectal cancer: a multicenter retrospective study

**DOI:** 10.1186/s12885-023-10640-9

**Published:** 2023-02-27

**Authors:** Qianhao Meng, Jian Zhao, Yuanyuan Yu, Ke Wang, Jing Ren, Chang Xu, Yusheng Wang, Guangyu Wang

**Affiliations:** 1 Department of Gastrointestinal Medical Oncology, Harbin Medical University Cancer Hospital, 150 Haping Road, Harbin, 150040 People’s Republic of China; 2 Department of Digestive, Shanxi Province Cancer Hospital/ Shanxi Hospital Affiliated to Cancer Hospital, Chinese Academy of Medical Sciences/Cancer Hospital Affiliated to Shanxi Medical University, Taiyuan, Shanxi 030013 People’s Republic of China

**Keywords:** Advanced colorectal cancer, BRAF V600E mutation, Overall survival, Progression-free survival

## Abstract

**Background:**

Patients with V-Raf murine sarcoma viral oncogene homolog B1 (BRAF) V600E-mutated advanced colorectal cancer (CRC) have a poor prognosis, and treatment options that can improve outcome are still under investigation. The purpose of this study was to discuss the differences of overall survival (OS) and progression-free survival (PFS) between patients with BRAF V600E-mutated advanced CRC who were treated with chemotherapy alone and chemotherapy combined with targeted therapy in advanced first-line therapy.

**Methods:**

Grouping of 61 patients according to first-line treatment regimen (chemotherapy alone/chemotherapy combined with bevacizumab). Kaplan–Meier method and log-rank test were used to compare OS and PFS. Cox proportional hazards regression model was used to measure the risk of first-line medication therapies while correcting for confounding factors that may affect PFS and OS.

**Results:**

There was no significant difference in OS between patients treated with chemotherapy alone and those treated with chemotherapy combined with bevacizumab (*P =* 0.93; HR, 1.027; 95% CI, 0.555–1.901). Likewise, there was no significant difference in PFS between the two groups (*P =* 0.29; HR, 0.734; 95% CI, 0.413–1.304). Subgroup analysis showed that OS and PFS of different treatment regimens were not significantly different among subgroups. Multivariate analysis suggested that surgical treatment of primary tumor (*P =* 0.001; HR, 0.326; 95% CI, 0.169–0.631) and presence of liver metastasis (*P =* 0.009; HR, 2.399; 95% CI, 1.242–4.635) may serve as independent prognostic indicators in patients with BRAF-mutated advanced CRC. Surgical treatment of the primary tumor (*P =* 0.041; HR, 0.523; 95% CI, 0.280–0.974) was significantly associated with PFS too.

**Conclusion:**

For patients with BRAF V600E-mutated advanced CRC, chemotherapy alone did not differ significantly in OS and PFS compared with chemotherapy + bevacizumab for advanced first-line therapy. Chemotherapy combined with targeted therapy did not render a survival benefit to these patients, demonstrating that the importance of developing new treatment options for this population.

**Supplementary Information:**

The online version contains supplementary material available at 10.1186/s12885-023-10640-9.

## Introduction

Colorectal cancer (CRC) is one of the most common types of malignant tumor and its incidence rate ranks third globally. Furthermore, the mortality rate of CRC is second only to lung cancer [1]. In recent years, with economic development and changes to both lifestyle and diet, both the morbidity and mortality rates of CRC in China have increased. In 2020, new cases of CRC in China accounted for 28.8% of all CRC cases worldwide and the number of CRC-related deaths in China accounted for 30.6% of global CRC-related deaths [2]. Surgery is the primary treatment strategy for patients diagnosed with early-stage CRC and the curative rate is high. However, 25% of patients are diagnosed with advanced or metastatic disease [3]. Unfortunately, this group of patients is more difficult to treat and has a poor prognosis, with a 5-year survival rate of ~ 14% [4]. Patients identified as having V-Raf murine sarcoma viral oncogene homolog B1 (BRAF) mutations via genetic testing, have a poorer prognosis [5–9]. BRAF encodes a serine/threonine protein kinase of the RAF family and is involved in the regulation of the RAS-RAF-MEK-ERK signaling pathway [10]. This signaling pathway serves a role in tumor growth and progression, including proliferation, angiogenesis, invasion and metastasis [11]. BRAF mutations lead to the overexpression of this pathway, which results in uncontrolled tumor growth [12]. The BRAF mutation is carried by 5.4–6.7% of Asian patients with CRC [13]. It mainly arises from serrated adenoma and is present in patients with right-sided colon cancer or who are females or who possess microsatellite instability-high [14, 15]. Among patients with BRAF mutations, the BRAF V600E mutation is the most common, accounting for ~ 80% [16].

At present, according to the 2021 Chinese Society of Clinical Oncology (CSCO) guidelines, the first-line of treatment for patients with BRAF-mutated advanced CRC is recommended to be FOLFOX/FOFIRI/XELOX ± bevacizumab for grade I, and FOLFOXIRI ± bevacizumab for grade II. The recommended second-line treatment for grade I CRC is chemotherapy ± bevacizumab [17]. Furthermore, in addition to bevacizumab treatment, research on other targeted drugs is also underway. Previous studies reported that epidermal growth factor receptor (EGFR)-targeted therapy does not have high single-agent activity in patients with BRAF-mutated metastatic (m)CRC [18, 19]. Similarly, BRAF inhibitor monotherapy is also ineffective in BRAF-mutated CRC, although BRAF inhibitors have shown striking efficacy in BRAF-mutant melanoma [20]. One reason for this is that following BRAF inhibition in CRC, negative feedback activates EGFR and the tumor-promoting signal detours the BRAF bypass to activate the downstream protein kinases MEK and ERK, which results in drug resistance [21]. However, the combination of a BRAF inhibitor and a MEK inhibitor produces more potent and sustained inhibition of MAPK signaling in BRAF mutant CRC cells, which leads to improved efficacy [22]. Moreover, it has previously been reported that a BRAF + EGFR inhibitor combination improves efficacy compared with a BRAF inhibitor alone [21, 23].

The ANCHOR-CRC study (ClinicalTrials.gov identifier: NCT03693170) has introduced targeted-drug triple therapy as a first-line treatment for patients with mCRC with BRAF V600E mutations. Based on the SWOG 1406 study, the VIC regimen (cetuximab + irinotecan + vemurafenib) is recommended by CSCO guidelines for second- and third-line treatment of BRAF V600E-mutated mCRC [24]. Moreover, based on the updated survival analysis of the BEACON study, the dual-target regimen has become the new standard of retreatment for relapsed BRAF V600E-mutated mCRC [25]. Various studies have demonstrated that chemotherapy tolerance and efficacy are poor in patients with advanced CRC with BRAF-mutations. For later-stage therapy, targeted combination therapy has become the standard treatment option. However, whether targeted combination therapy can be used as first-line treatment remains to be unclear and its application in advanced first-line treatment is still under intense discussion.

The aim of the present study was to discuss the differences between overall survival (OS) and progression-free survival (PFS) in patients with BRAF V600E-mutated advanced CRC who were treated with chemotherapy alone versus chemotherapy combined with a targeted therapy. The results of the present study have provided guidance for targeted combination therapy as an advanced first-line treatment.

## Methods

### Patients

This present retrospective analysis used the survival data of patients with BRAF-mutated advanced CRC who received first-line treatment at the Harbin Medical University Cancer Hospital and Shanxi Province Cancer Hospital from March 2015 to August 2021. The patients were divided into two groups according to the first-line regimen (chemotherapy-only/chemotherapy + bevacizumab). The present study was approved by the Ethics Committees of Harbin Medical University Cancer Hospital and Shanxi Province Cancer Hospital. Patient data remained confidential. The present study complies with The Declaration of Helsinki.

### Inclusion criteria

The inclusion criteria for the present study were as follows: (1) CRC was diagnosed by preoperative endoscopic biopsy or postoperative pathology; (2) genetic testing revealed a BRAF V600E mutation; and (3) all patients received advanced first-line therapy with chemotherapy/chemotherapy + bevacizumab.

### Exclusion criteria

The exclusion criteria for the present study were as follows: (1) Patients with a history of other malignancies; and (2) the time from the end of postoperative adjuvant chemotherapy to recurrence or metastasis was less than 6 months.

### Clinicopathological characteristics

Clinical data, including age, gender, Eastern Cooperative Oncology Group (ECOG) score, primary tumor site, histological grade, number of metastatic sites, primary tumor surgery, mismatch repair (MMR) status, intestinal obstruction status, liver metastasis, lung metastasis, peritoneal metastasis and distant lymph node metastasis were recorded.

### Follow-up

Patient follow-up information was obtained from hospital records or from the patients and their families. OS was determined as the primary endpoint of the study and PFS, objective response rate (ORR) and disease control rate (DCR) were determined as secondary endpoints. OS is defined as the time from discovery of recurrence or metastasis with no chance of cure or transformation until death from any cause. PFS is defined as the time from discovery of recurrence or metastasis with no chance of cure or transformation until progression. For patients who lost follow-up, we recorded their final follow-up time. Patients undergo the CT scan every 1.5–2 months during treatment. ORR was defined as the proportion of patients with the best complete response (CR) and partial response (PR), and DCR was defined as the proportion of patients with the best CR, PR and stable disease (SD) according to RECIST1.1 criteria. All patients were followed up for at least three years.

### Statistical analysis

Patients were grouped according to first-line treatment regimens (chemotherapy alone/chemotherapy + bevacizumab). The chi-square test and Fisher’s exact test were used to analyze baseline characteristics. Univariate analysis was performed using the Kaplan–Meier method and log-rank test. OS and PFS survival curves were plotted and compared. The Cox proportional hazards regression model was used to assess the relationship between first-line medication status and survival prognosis, and hazard ratios (HR) and corresponding 95% confidence intervals (CI) for OS and PFS were estimated. The confounding factors that may affect OS and PFS were also analyzed. *P <* 0.05 was considered to indicate a statistically significant difference. Statistical analysis was performed in July 2022 using SPSS (version 25.0) software (IBM Corp.) and R software (version 4.2.0).

## Results

From 2015 to 2021, a total of 61 patients met the inclusion criteria for the present study. Of these patients, the median age was 59 (range, 28–81) years and 34 patients (55.7%) were male. The median follow-up time was 39.2 months. According to the last patient follow-up (June 22, 2022), in terms of OS, 41 patients died, seven patients survived and 13 patients lost contact and were not included in the follow-up. For PFS, 47 patients progressed, eight patients did not progress and six patients lost contact and were not included in the follow-up. For patients who survived or were lost to follow-up, the time of last follow-up was recorded as the OS. For deceased patients who did not experience a PFS, the time of death was recorded for the PFS. For patients who survived or were lost to follow-up, whereby the PFS had not been recorded, the final follow-up time was recorded as the PFS.

**Fig. 1:**
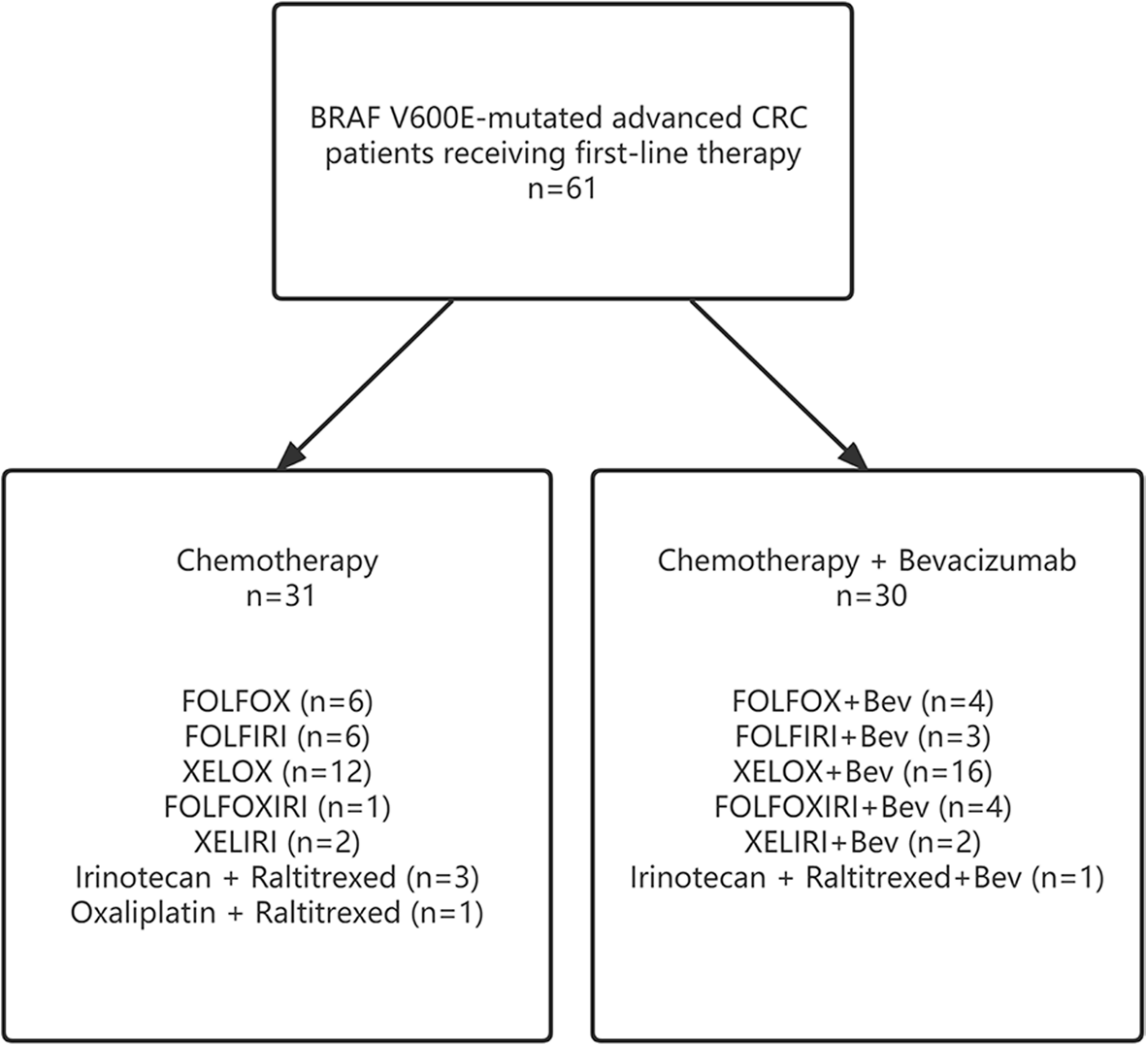
Flowchart of patient treatment

The patients were divided into two groups according to the first-line regimen (chemotherapy-only/chemotherapy + bevacizumab). Among them, there were 31 patients in the chemotherapy-only group and 30 in the chemotherapy + bevacizumab group. The median age of patients in the chemotherapy-only group was 61, ranging from 28 to 81. The median age of patients in the chemotherapy + bevacizumab group was 58.5 years, with a range of 32–80 years. Among 61 patients, 31 patients (50.8%) received second-line treatment, including 14 in the chemotherapy-only group and 17 in the chemotherapy + bevacizumab group. 17 patients (27.9%) received third-line and posterior-line treatment, including 8 in chemotherapy-only group and 9 in chemotherapy + bevacizumab group. The first-line chemotherapy regimens included XELOX/FOLFOX/FOLFIRI/XELIRI/FOLFIRINOX/irinotecan + raltitrexed/oxaliplatin + raltitrexed (Fig. 1). Baseline characteristics of patients in the two groups were similar (Table 1). There was no statistically significant difference between the two treatment groups for age, gender, ECOG score, primary tumor site, histological grade, the number of metastatic sites, primary tumor surgery, MMR status, intestinal obstruction, liver metastasis, lung metastasis, peritoneal metastasis and distant lymph node metastasis (*P >* 0.05).

**Table 1:** Patient and tumor characteristics in patients with BRAF-mutated

Characteristic	Chemo(*n =* 31)	Chemo + targeted therapy(*n =* 30)	χ2	*P*-value
Age				
< 65 years	20	21	0.208	0.648
≥ 65 years	11	9		
Gender				
Male	17	17	0.021	0.886
Female	14	13		
Ecog				
< 2	27	27	0.126	0.722
≥ 2	4	3		
Tumor site				
Multi-sides	2	0	4.173	0.206
Right colon	13	9		
Left colon	9	8		
Rectum	7	13		
Histologic grade				
Missing	4	3	0.283	1
Low grade	11	12		
High grade	16	15		
Number of metastatic sites				
Single	17	14	0.407	0.523
Multiple	14	16		
MMR Status				
Missing	10	8	3.227	0.229
dMMR	3	0		
pMMR	18	22		
Primary tumor surgery				
No	10	16	2.769	0.096
Yes	21	14		
Intestinal obstruction				
No	29	27	0.109	0.742
Yes	5	3		
Liver metastases				
No	14	14	0.014	0.906
Yes	17	16		
Lung metastases				
No	19	22	1.003	0.316
Yes	12	8		
Peritoneal metastasis				
No	27	24	0.162	0.687
Yes	4	6		
Distant lymph node metastases				
No	24	19	1.454	0.228
Yes	7	11		

**Fig. 2:**
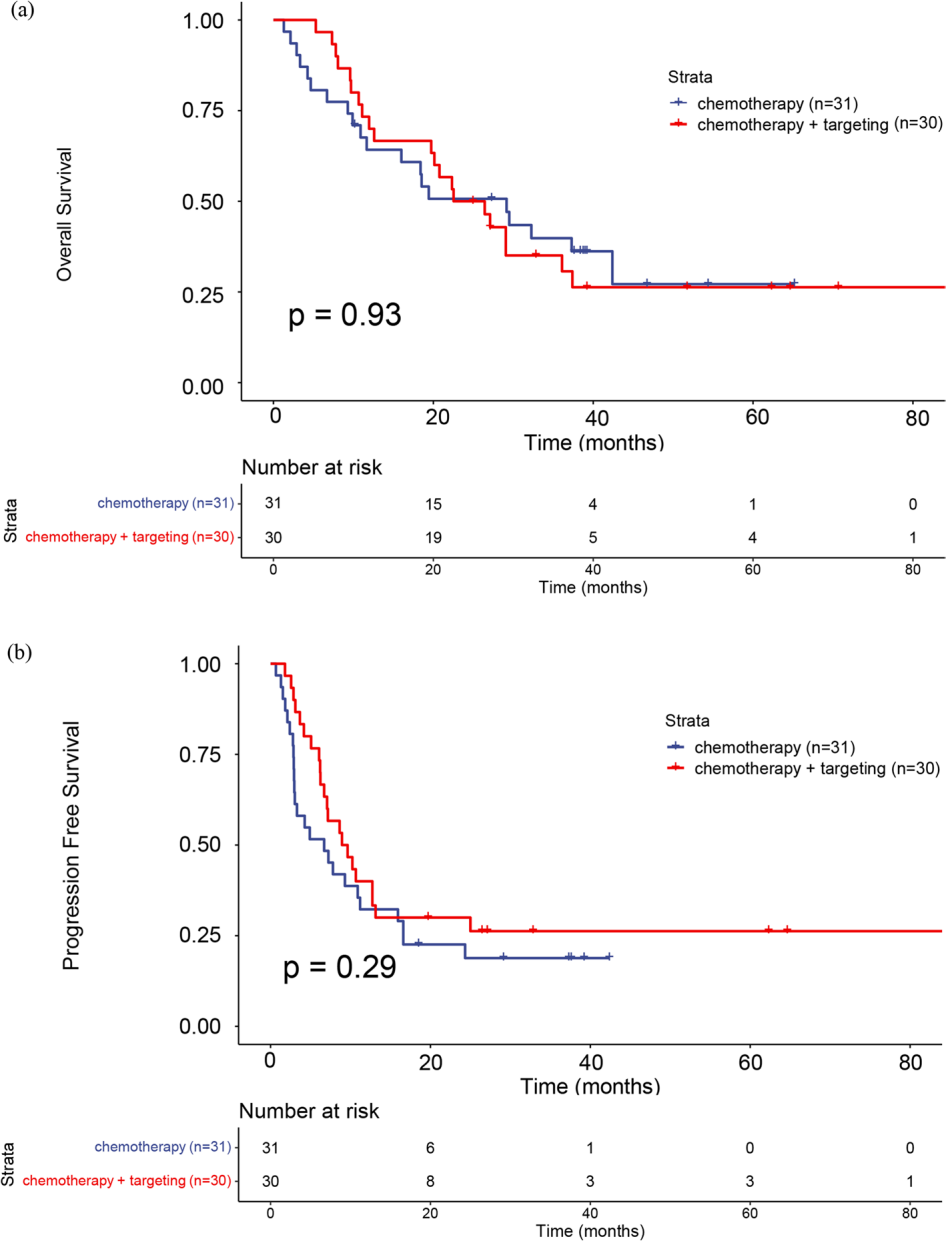
**a** Kaplan–Meier survival analysis for OS (**b**) Kaplan–Meier survival analysis for PFS

The survival outcomes between the two groups of patients were compared. For the primary endpoint, the 3-year OS rate was 39.8% in the chemotherapy-only group, whereas the OS rate was 35.1% in the chemotherapy + bevacizumab group. The median OS was 29.2 months in the chemotherapy-only group but was 24.5 months in the chemotherapy + bevacizumab group. Compared with chemotherapy alone, patients in the chemotherapy + bevacizumab group did not gain a significantly longer OS (*P =* 0.93; HR, 1.027; 95% CI, 0.555–1.901) (Fig. 2a).

For the secondary study endpoint, the 1-year PFS rates were 32.3 and 40% in the chemotherapy-only group and the chemotherapy + bevacizumab group, respectively. The median PFS was 6.7 months in the chemotherapy-only group and 9.28 months in the chemotherapy + bevacizumab group. Compared with the chemotherapy-only group, patients in the chemotherapy + bevacizumab group had no significant prolongation of PFS (*P =* 0.29; HR, 0.734; 95% CI, 0.413–1.304) and the difference was not statistically significant (Fig. 2b). In the chemotherapy-only group, the ORR was 14.3% and the DCR was 52.4%. In the chemotherapy + bevacizumab group the ORR was 24.1% and the DCR was 89.7%. There was no significant difference in ORR between the two groups (*P =* 0.616). However, the DCR of the chemotherapy + bevacizumab treatment group was significantly improved compared with the chemotherapy-only group (*P =* 0.003, Table 2).

**Table 2:** Response of patients with measurable disease

Response	All	Chemotherapy	Chemo + bevacizumab
	*n =* 61	*n =* 31	*n =* 30
PR	10	3	7
SD	27	8	19
PD	13	10	3
Missing	11	10	1
ORR	20%	14.30%	24.10%
DCR	74%	52.40%	89.70%

**Fig. 3:**
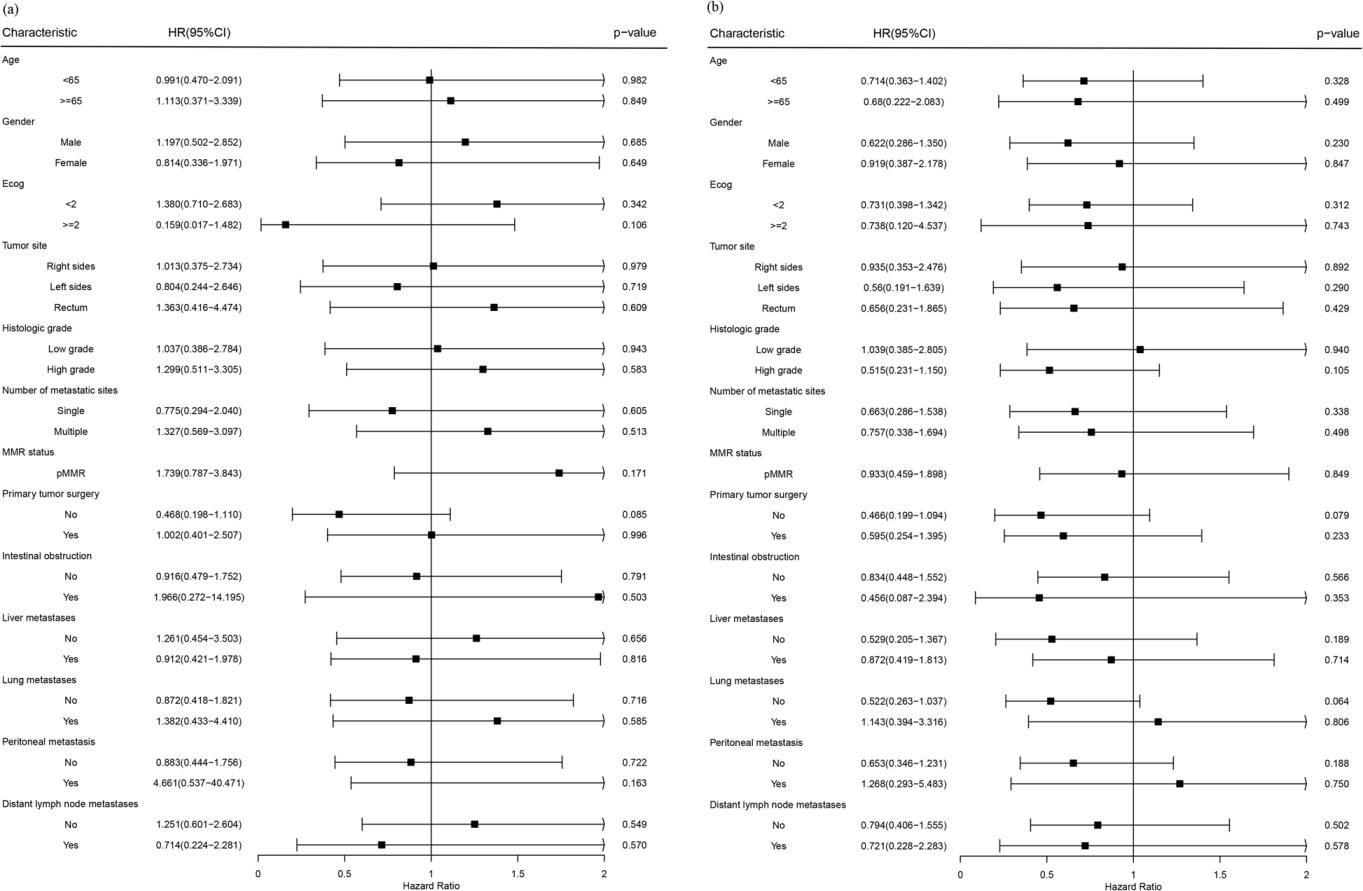
**a** Subgroup analyses of OS (**b**) Subgroup analyses of PFS

Subgroup analysis demonstrated that there was no statistically significant difference in OS and PFS between the two treatment groups in each subgroup of patients with BRAF-mutated advanced CRC. In terms of first-line treatment, chemotherapy alone and chemotherapy + bevacizumab displayed no significant difference in efficacy among subgroups (*P >* 0.05) (Fig. 3a and b).

Univariate analysis demonstrated that the number of metastases (*P =* 0.030; HR, 2.003; 95% CI, 1.071–3.745), the surgical treatment of the primary tumor (*P <* 0.001; HR, 0.285; 95% CI, 0.152–0.537) and liver metastasis (*P =* 0.006; HR, 2.480; 95% CI, 1.302–4.724) were significantly associated with OS. Significant factors (*P <* 0.05) were included in the multivariate analysis. The multivariate analysis demonstrated that surgical treatment of the primary tumor (*P =* 0.001; HR, 0.326; 95% CI, 0.169–0.631) and liver metastasis (*P =* 0.009; HR, 2.399; 95% CI, 1.242–4.635) could potentially be used as independent prognostic indicators of OS in patients with BRAF-mutated advanced CRC (Table 3).

**Table 3:** Univariate and multivariate analysis of OS

OS				
Characteristic	Univariable analysis		Multivariable analysis	
	HR (95%CI)	*P*-value	HR (95%CI)	*P*-value
Age(< / ≥ 65 years)	0.882(0.457–1.705)	0.710		
Gender(Male/Female)	1.108(0.600–2.046)	0.743		
Ecog(< 2/ ≥ 2)	0.947(0.368–2.437)	0.909		
Tumor site(Multi-sides/Right colon /Left colon/Rectum)	0.920(0.666–1.271)	0.613		
Histologic grade(Low grade/High grade)	0.954(0.486–1.873)	0.891		
Number of metastatic sites(Single/Multiple)	2.003(1.071–3.745)	0.030*	1.603(0.842–3.051)	0.151
MMR Status(dMMR/pMMR)	1.367(0.323–5.782)	0.671		
Primary tumor surgery(No/Yes)	0.285(0.152–0.537)	< 0.001*	0.326(0.169–0.631)	0.001*
Intestinal obstruction(No/Yes)	0.655(0.233–1.841)	0.422		
Liver metastases(No/Yes)	2.480(1.302–4.724)	0.006*	2.399(1.242–4.635)	0.009*
Lung metastases(No/Yes)	0.839(0.427–1.648)	0.610		
Peritoneal metastasis(No/Yes)	1.162(0.536–2.518)	0.704		
Distant lymph node metastases(No/Yes)	0.907(0.462–1.782)	0.778		
First-line medication(Chemo/Chemo + targeted therapy)	1.027(0.555–1.901)	0.932		

Univariate analysis also demonstrated that primary tumor surgery (*P =* 0.006; HR, 0.438; 95% CI, 0.243–0.790) and liver metastases (*P =* 0.018; HR, 2.058; 95% CI, 1.132- 3.742) were significantly associated with PFS. Factors with *P <* 0.05 were included in the multivariate analysis, which demonstrated that surgical treatment of the primary tumor (*P =* 0.041; HR, 0.523; 95% CI, 0.280–0.974) was significantly associated with PFS (Table 4).

**Table 4:** Univariate and multivariate analysis of PFS

PFS				
Characteristic	Univariable analysis		Multivariable analysis	
	HR (95%CI)	*P*-value	HR (95%CI)	*P*-value
Age(< / ≥ 65 years)	0.561(0.295–1.064)	0.077		
Gender(Male/Female)	1.192(0.670–2.121)	0.551		
Ecog(< 2/ ≥ 2)	1.005(0.397–2.546)	0.991		
Tumor site(Multi-sides/Right colon /Left colon/Rectum)	0..941(0.692–1.281)	0.700		
Histologic grade(Low grade/High grade)	1.350(0.718–2.537)	0.351		
Number of metastatic sites(Single/Multiple)	1.445(0.814–2.564)	0.209		
MMR Status(dMMR/pMMR)	4.070(0.553–29.943)	0.168		
Primary tumor surgery(No/Yes)	0.438(0.243–0.790)	0.006*	0.523(0.280–0.974)	0.041*
Intestinal obstruction(No/Yes)	1.657(0.739–3.716)	0.221		
Liver metastases(No/Yes)	2.058(1.132–3.742)	0.018*	1.677(0.890–3.157)	0.109
Lung metastases(No/Yes)	0.667(0.355–1.254)	0.208		
Peritoneal metastasis(No/Yes)	1.813(0.840–3.915)	0.130		
Distant lymph node metastases(No/Yes)	0.702(0.364–1.353)	0.291		
First-line medication(Chemo/Chemo + targeted therapy)	0.734(0.413–1.304)	0.291		

## Discussion

In advanced CRC, systemic chemotherapy ± bevacizumab has been the cornerstone of therapy in patients with BRAF V600E mutations [26]. However, such patients respond poorly to conventional chemotherapy regimens [27]. In the 2015 TRIBE study, subgroup results of BRAF mutations demonstrated that FOLFOXIRI + bevacizumab provided survival benefits for patients compared to FOLFIRI + bevacizumab (OS, 19.0 months vs 10.7 months, respectively) [28]. However, the results of subsequent clinical studies were not satisfactory. The BRAF mutation subgroup resulted in the TRIBE2 study exhibiting no survival benefits from FOLFOXIRI + bevacizumab treatment [29]. In 2020, a meta-analysis of five randomized trials comparing FOLFOXIRI + bevacizumab to doublet chemotherapy + bevacizumab failed to show any advantage of FOLFOXIRI + bevacizumab in subgroup analyses [30]. In terms of first-line treatment for BRAF V600E-mutated mCRC, there is insufficient evidence to suggest that a triple cytotoxic regimen has significant benefits compared with doublet chemotherapy. This implies that chemotherapy may not work well for such patients and suggests that focus should be given to targeted drugs.

At present, multiple clinical studies for targeted drugs for patients with BRAF V600E-mutated mCRC are being performed. The results of the SWOG S1406 study recommended irinotecan + cetuximab + vemurafenib for second-line and later treatment of patients with RAS wild-type/BRAF V600E mutations [24]. The BEACON study, presented by Tabernero J, was one of the first studies to suggest the use of second-line chemotherapy-free targeted therapy for these patients [25]. The dual-target regimen (encorafenib + cetuximab) and the triple-target regimen (encorafenib + binimetinib + cetuximab) had similar OS and PFS rates and exhibited significantly improved OS and PFS compared with chemotherapy alone (irinotecan + cetuximab or FOLFIRI + cetuximab). However, the dual-target regimen had a lower incidence of grade 3 adverse events [25]. The 2021 National Comprehensive Cancer Network guidelines [26] recommend a BRAF inhibitor + cetuximab for second-line and later-stage treatment of patients with the RAS wild-type/BRAF V600E mutation. Furthermore, BRAF inhibitor + cetuximab + MEK inhibitor can be considered for patients with extensive metastatic sites and heavier tumor burden [31].

For later treatment of BRAF-mutated populations, evidence indicates that targeted combination therapy, with no chemotherapy component, can lead to the longer survival of patients with a better quality of life. However, for first-line treatment, there is still lack of evidence to support targeted combination therapy without chemotherapy. Therefore, in the present study, the efficacy of chemotherapy-only and chemotherapy + bevacizumab first-line treatment in patients with advanced CRC with BRAF V600E mutation, was compared. Furthermore, the prognosis of the patients receiving the different treatment regimens was also compared. Data were extracted from hospital records using strict inclusion/exclusion criteria. Data analysis demonstrated that compared with chemotherapy alone, chemotherapy + bevacizumab did not exhibit a statistically significant increase in survival time. In subgroups, the two treatment groups also showed no significant survival differences. For patients whose primary tumor has not undergone surgery, there was a trend of benefit with chemotherapy + bevacizumab. However, there was no statistically significant difference (OS, *P =* 0.085; PFS, *P =* 0.079). These results therefore indicated that in first-line treatment, chemotherapy + bevacizumab for the treatment of patients with advanced CRC with BRAF V600E mutation, has a similar prognosis to chemotherapy alone, and neither may markedly prolong the survival of the patients. It can therefore be hypothesized that there are better advanced first-line treatment regimens than the two applied in the present study.

In the ANCHOR study (ClinicalTrials.gov identifier: NCT03693170), patients with the BRAF V600E mutation were treated with triple-targeted therapy consisting of encorafenib + cetuximab + binimetinib as first-line treatment. The study met its primary endpoint with a final confirmed ORR of 47.8%, which was markedly higher than standard chemotherapy. The DCR was 88%, the median PFS was 5.8 months and the median OS was 17.2 months [32]. However, this study was not a randomized study, so it can therefore not be confirmed that this regimen can replace standard treatment. The ongoing BREAKWATER study (ClinicalTrials.gov identifier: NCT04607421) is currently evaluating the efficacy of encorafenib + cetuximab with or without chemotherapy versus standard therapy (FOLFOX/FOLFIRI/FOLFOXIRI, etc. ± anti-vascular endothelial growth factor antibody) as first-line treatment. If positive results are obtained from this study, it could change the standard for first-line treatment.

The 61 patients in the present study had a longer median OS (29.2/24.5 months) than other patients with BRAF-mutated mCRC patients on other studies [33–35]. This may suggest that in ‘real-world’ studies, according to the ‘BRAF BeCool’ score, cases in the low/intermediate-risk category are prevalent [34]. However, there are still relatively long median OS results [36]. In BRAF-mutated CRC, surgical resection of the primary tumor and liver metastasis are closely related to patient survival [34, 37]. This is consistent with the data obtained from the present study.

In stage I-III colorectal cancer, a combination of molecular markers, tumor location with the other clinical-pathological variables and microsatellite status may be useful predictors [38].Previous studies have clarified the association of MSI/BRAF combination subgroup on clinical outcomes in CRC, supporting the prognostic role of MSI/BRAF combined detection in CRC [39]. Studies have shown that BRAF mutations significantly shorten the survival of mCRC patients with MMR-deficient (dMMR) [40]. BRAF-mutated proximal colon adenocarcinomas with proficient DNA mismatch repair have a dismal prognosis with an aggressive clinical course [41]. Given the association between BRAF mutation status and MMR status [42] and the recognized prognostic value of MMR status, it is important to consider MMR status when assessing the relationship between BRAF status and survival. Our study concluded that among 40 BRAF-mutated patients in MMR-proficient (pMMR) status, there was no significant difference in survival between chemotherapy + bevacizumab group and chemotherapy-only group. However, in our study, there were only 3 patients with dMMR status, which is a very small number. This may be due to the fact that the study population in our study was advanced CRC, and dMMR status was mainly present in CRC with stage I-III [43]. Therefore, the efficacy of chemotherapy or chemotherapy + bevacizumab in BRAF-mutated tumors with dMMR status has not been adequately evaluated. We also failed to assess the effect of MMR status in patients with BRAF mutations accurately.

The limitations of the present study should be noted when analyzing the results. First, this was a retrospective study and the data collected are inevitably biased. Second, safety data concerning patient treatment was not available. Evaluation of adverse effects of chemotherapy/chemotherapy + bevacizumab in patients was lacking. Third, the effect of treatment regimens on BRAF-mutant tumors with dMMR status and the effect of MMR status on BRAF-mutated patients failed to assess. Therefore, in order to further verify the experimental results, it will be necessary to perform a large-scale prospective clinical randomized controlled trial. Despite the inevitable limitations, the present study still provides a reference for clinicians to determine the best treatment options and also provides the basis for follow-up research.

## Conclusions

The results of the present study demonstrated that for patients with BRAF V600E-mutated advanced CRC, chemotherapy alone did not differ significantly in OS and PFS compared with chemotherapy + bevacizumab for advanced first-line therapy. The most commonly used drug regimen-chemotherapy combined with a targeted therapy did not render survival benefits to these patients. These results therefore demonstrated the importance of developing new treatment options for this population. These data have provided a reference point for the progression of follow-up research.

## Supplementary Information

**Additional file 1.** STROBE Statement—checklist of items that should be included in reports of observational studies.

## Data Availability

The datasets used and/or analysed during the current study are available from the corresponding author on reasonable request.

## Abbreviations

BRAF: V-Raf murine sarcoma viral oncogene homolog B1
CI: Confidence intervals;
CR: Complete response
CRC: Colorectal cancer
DCR: Disease control rate
dMMR: Mismatch repair-deficient
ECOG: Eastern Cooperative Oncology Group
EGFR: Epidermal Growth Factor Receptor
HR: Hazard ratios
MMR: Mismatch repair
ORR: Objective response rate
OS: Overall survival
PFS: Progression-free survival
pMMR: Mismatch repair-proficient
PR: Partial response
SD: Stable disease
